# Role of wind in alteration of hilltop airborne bacterial communities enriched with pathogens over the Eastern Himalayas in India

**DOI:** 10.1128/aem.02187-25

**Published:** 2025-12-18

**Authors:** Shahina Raushan Saikh, Antara Pramanick, Md Abu Mushtaque, Sanat Kumar Das

**Affiliations:** 1 Department of Physical Sciences, Bose Institute 30141, Kolkata, West Bengal, India; 2 Department of Life Science and Bio-technology, Jadavpur University 30167, Kolkata, West Bengal, India; 3 Department of Microbiology, University of Calcutta 30163, Kolkata, West Bengal, India; Colorado School of Mines, Golden, Colorado, USA

**Keywords:** airborne bacteria, pathogen, Eastern Himalayas, meteorology, wind

## Abstract

Airborne microorganisms play a significant role in atmospheric processes and public health, yet their variations over high-altitude regions are underexplored. To investigate the meteorological influence and role of transport patterns on airborne microorganisms, we analyzed DNA sequencing of bacterial population collected from ambient atmosphere during 2022–2023 over Darjeeling (27.03°N, 88.26°E; 2,200 m amsl), an Eastern Himalayan hilltop site, and categorized as winter (dry: cold, stable), pre-monsoon (semi-dry: warm, transitional), monsoon (wet: humid, rainy), and post-monsoon (semi-wet: cooler, cloudy) seasons. Back-trajectory analysis showed air masses from the western Indo-Gangetic Plain during pre-monsoon and from the Bay of Bengal during monsoon, while winter and post-monsoon air masses were predominantly regional hilly winds. Significant seasonal variability in airborne bacterial populations was noticed over the Eastern Himalayas, with highest abundance and diversity in pre-monsoon (cell count = 5.8 ± 1.9 × 10^5^ m^−3^, operational taxonomic units = 597 ± 343, genera = 188 ± 76, Shannon = 4.1 ± 1.0) due to continental wind transport and particulate matter influx. About one-fourth of airborne bacterial genera were persistent in all seasons, representing background Himalayan hilltop airborne bacterial population. Unique season-specific genera are prominent in pre-monsoon (15%), followed by post-monsoon (7%), monsoon (6%), and winter (4%), indicating significant enrichment of airborne bacteria due to the influence of wind. Positive correlations with wind speed (*r* = 0.57, *P* < 0.05), temperature (*r* = 0.50, *P* < 0.05), and PM_2.5_ (r = 0.84, *P* < 0.001) indicate the role of meteorological parameters in shaping airborne bacterial population. Human pathogens like *Acinetobacter, Staphylococcus,* and *Corynebacterium,* responsible for gastroenteritis and respiratory, skin, and urinary tract infections, highlight potential health risks and the importance of integrating atmospheric biological data and meteorological modeling into public health strategies over Eastern Himalayan region.

**IMPORTANCE:**

Airborne microorganisms play an important role in atmospheric processes, ecosystem functioning, and human health. However, their dynamics in high-altitude regions are poorly characterized. The present study provides the first comprehensive seasonal assessment of Eastern Himalayan airborne bacterial diversity and abundance, revealing strong meteorological control, particularly wind patterns and particulate matter, on airborne bacterial loading and community composition. Identification of opportunistic pathogenic bacterial genera across all seasons raises concerns about potential health impacts, especially in regions where population density and tourism are increasing. Our findings also highlight continental transport of airborne bacteria from distant source regions like the Indo-Gangetic Plain, suggesting airborne bacterial influx. By integrating atmospheric biological data with air-mass back-trajectory simulation, the present study highlights valuable insights into how wind influences Himalayan airborne bacterial community. These insights are essential for developing airborne bacterial forecasting tools and public health strategies in vulnerable hilltop atmospheres that undergo rapid environmental change.

## INTRODUCTION

Airborne microorganisms are omnipresent in the atmosphere, existing at a density of 10^3^–10^7^ cells/m^3^ (1–3), with multiple sources such as soil, forests, deserts, agriculture, flora, fauna, composts, industrial and hospital waste, sediments, etc. (4–6). They can survive under adverse circumstances, including nutritional deficiency, UV radiation, desiccation, fluctuations in temperature and pH, and oxidative stress (4, 7). Airborne microorganisms are metabolically active even at high altitudes of 20 km above ground (8–11). Viable bacteria and fungi have even been recovered at 48 km–70 km, transported by strong convection during dust storms (12), and show greater resistance to UV and freeze–thaw stress than near-surface species (12, 13). In addition, these microbes serve as disease vectors (14–16), further increasing human health risks (17) by affecting respiratory organs (18) and causing skin infections (19). Airborne microorganisms disperse through global wind patterns on inter- and intra-continental scales (3, 20–23), often attached to airborne particulate matter, and can rapidly spread from their source to downwind regions (18, 24, 25). Examples include Saharan Desert dust transport (3), Asian dust events increasing microbial diversity in the North American highlands (2.7 km amsl) (26), and Gobi Desert plumes carrying airborne bacteria to Japan and Korea (27).

Atmospheric microorganisms demand in-depth investigations in high-altitude regions to understand ecological and meteorological influences on their dynamics and dispersion over long distances (28). Asian dust events can lift large amounts of soil microorganisms to high altitudes, which can alter regional background microbial populations (18, 28, 29). A few studies on airborne microorganisms have been conducted at different altitudes and high-elevation sites, like New Mexico and California, USA (9); Colorado, USA (30); Spain (31); and Tibet, China (32). Despite the global importance of the Himalayan region as a climatic and ecological barrier, detailed investigations of airborne microbial communities in the Eastern Himalayas remain underexplored. The Eastern Himalayas represent a transitional zone where natural ecosystems coexist with dense urban settlements and high tourist influx. The high-altitude atmosphere of the Eastern Himalayas provides a unique opportunity to investigate the alterations in hilltop airborne bacterial communities as the Himalayan atmosphere experiences distinct air-mass influences from continental dry winds from western India and the Indo-Gangetic Plain (IGP) during the pre-monsoon season, and marine-influenced wet winds from eastern direction during the monsoon season (33). These seasonal shifts in atmospheric circulation make the Eastern Himalayas an exceptional sampling site to examine how large-scale air-mass transport and local topography alter airborne bacterial communities, with important impacts on regional ecology, climate, and public health.

In the present study, we analyze seasonal variability in the airborne bacterial communities at a high-altitude region over the Eastern Himalayas (2,200 m amsl). Using high-throughput sequencing of 16S rRNA V3-V4 regions, we analyzed 88 samples collected during all four seasons. We observed significant seasonal shifts in airborne bacterial abundance and composition, which are strongly influenced by wind speed (WS) and direction. We also determined the variation of pathogenic bacterial genera responsible for skin, respiratory, gastrointestinal tract (GIT), and oral infections in different seasons. The current study shows a comprehensive seasonal assessment of how meteorological conditions and air quality play a dynamic role in shaping airborne bacterial communities over the Eastern Himalayas.

### Sampling site description with geographical importance

**Fig 1:**
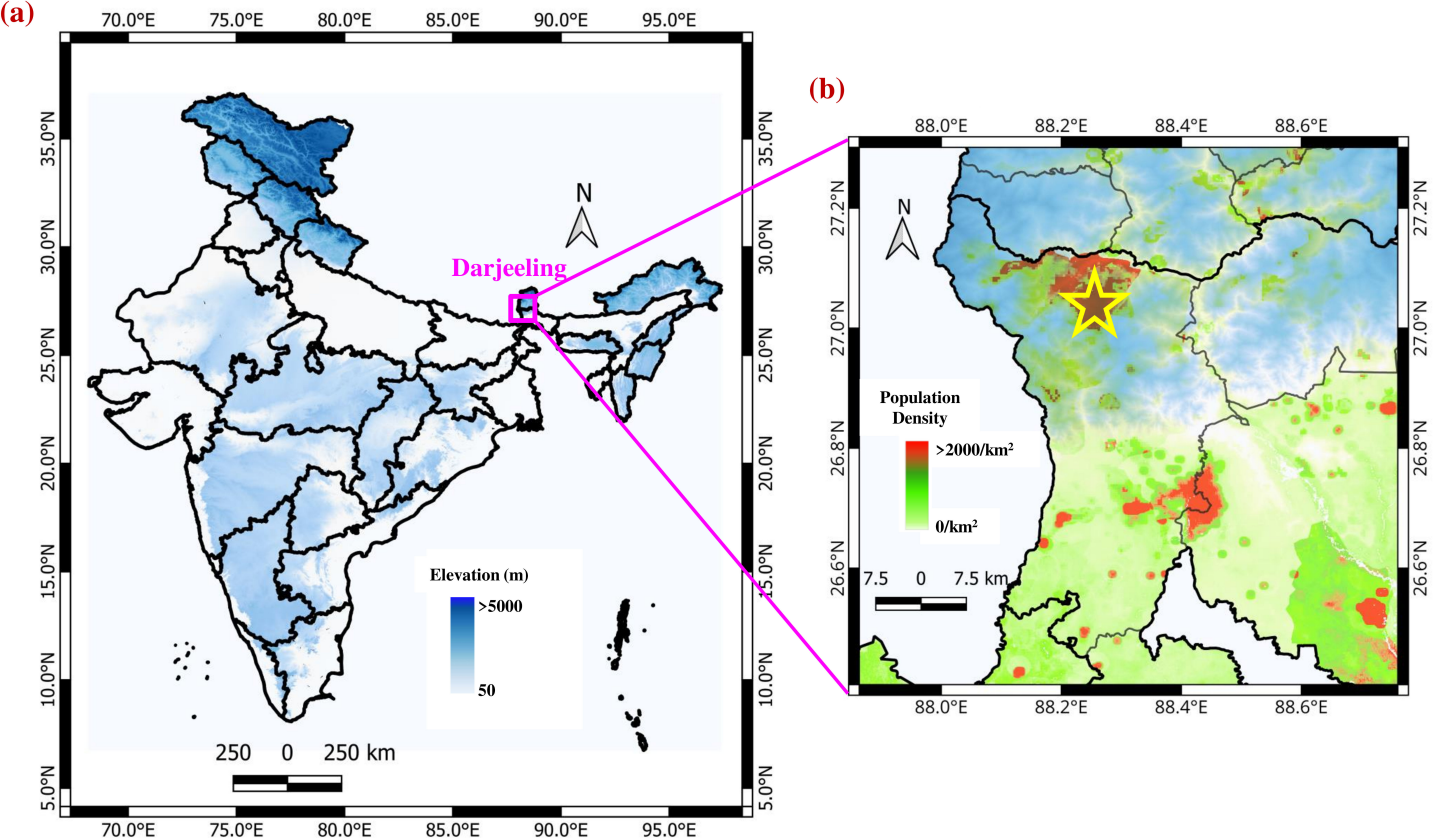
(**a**) Map of India with topographical elevation profile; the location of the sampling site is marked with a pink box in the Eastern Himalayas. (**b**) The measurement site in Darjeeling (27.03°N, 88.26°E) district is shown with a yellow star, which is located at an elevation of 2,200 m amsl. Population density is also represented according to WorldPop, 2018.

Regular airborne bacterial sampling was carried out from January 2022 to September 2023 within the campus of Bose Institute, situated at Darjeeling (27.03°N, 88.26°E, 2,200 m amsl) in West Bengal, India. The location of Darjeeling in India is marked with a pink box and shown in Fig. 1a, where colored filled contours represent the topographical variation over the Indian subcontinent. A star (yellow) shows the location of the sampling site in Darjeeling in Fig. 1b, where gridded 100 m resolution of WorldPop Population Count of India 2020 is overplotted (34). The measurement site was selected 15 m above the ground, on the rooftop of an institutional building surrounded by plantations. The plantations mainly consist of typical high-altitude Himalayan coniferous forests, and tea gardens are considered one of the tourist attractions in the city and are situated within a radius of ~10 km–12 km. Darjeeling is a popular tourist destination located in the Eastern Himalayas and represents a Himalayan urban atmosphere with a high population count of about 5,719,669 (Census 2011), as shown in Fig. 1b. The two most populous nations in the world, China and India, are divided by the intricate barrier formed by the Eastern Himalayan range. The sampling site is located approximately at an elevation of 200 m from the main township and about 70 km away from the nearest location of the IGP. The Eastern Himalayas have unique climatic characteristics due to their geographical location and broad range of altitudinal differences between 300 and 3,700 m amsl, having a typical monsoon climate with dry winters and wet summers. The Eastern Himalayas experience a strong wet monsoonal wind from June to August, coming from the Bay of Bengal (BoB), located roughly 600 km east of the measurement site, and a dry wind between March and May coming from Western India. Therefore, the climatic conditions of the Eastern Himalayas vary enormously depending on variations in rainfall, temperature, and air movement.

### Meteorological synopsis

Meteorological parameters (temperature, relative humidity (RH), WS, wind direction (WD), and solar radiation) were measured every minute, round the clock, from a portable automated weather station (Davis Vantage Pro2) installed on the rooftop of the measurement site. According to the Indian Meteorological Department, four distinct seasons are evident throughout the year over Darjeeling: winter (December–February), pre-monsoon (March–May), monsoon (June–August), and post-monsoon (September–November). To emphasize climatic characteristics, these seasons can be described more generally as dry, semi-dry, wet, and semi-wet, respectively, based on RH, temperature, solar radiation, and prevailing air masses.

**Fig 2:**
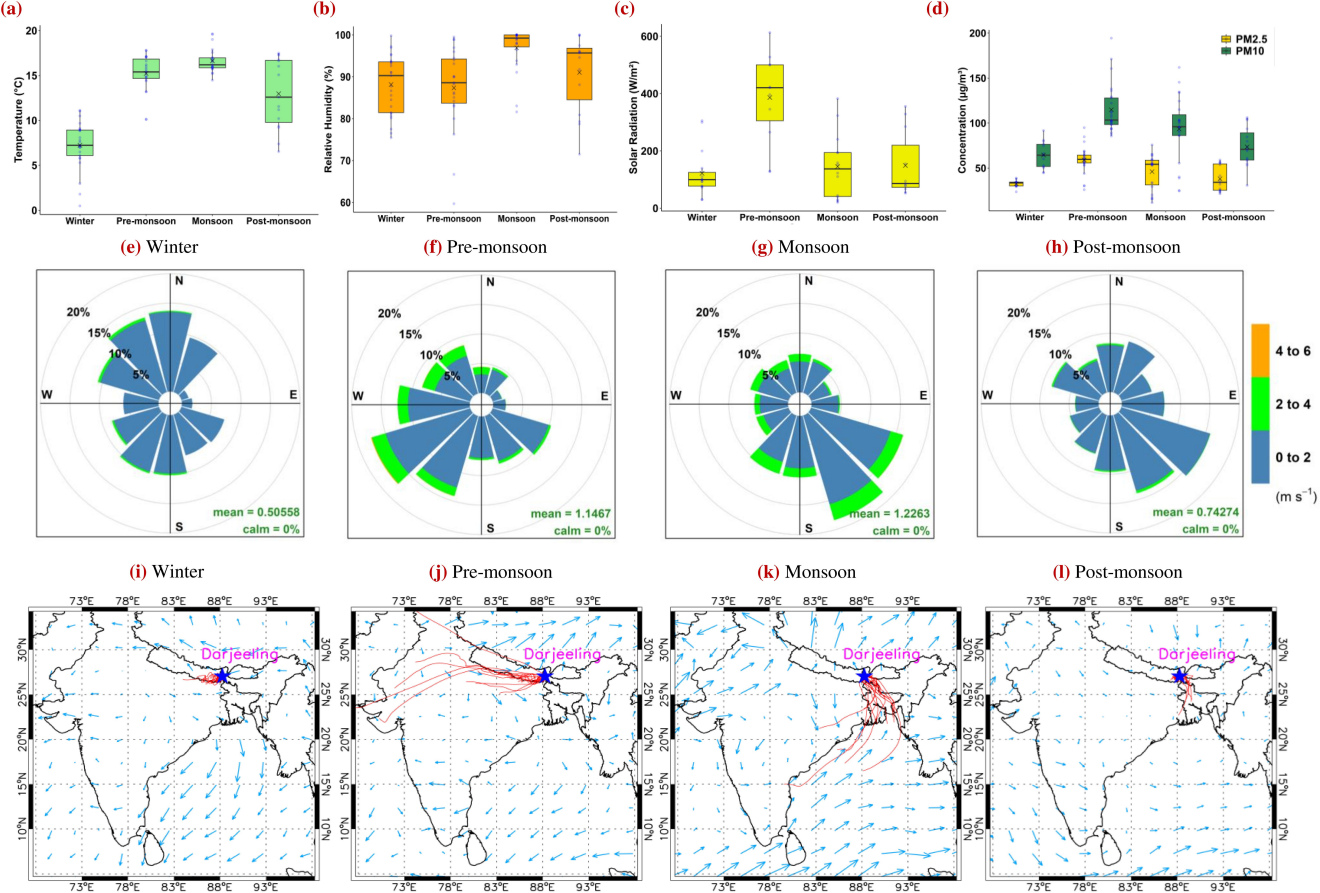
Box plot of seasonal variation of (**a**) RH, (**b**) temperature, (**c**) solar radiation, and (**d**) PM_2.5_ and PM_10_ over the Eastern Himalayas. The 95% confidence threshold (*P* < 0.05) was calculated for meteorological and air pollutant parameters to represent accurate mean with ±1σ standard deviation (vertical bars). Wind rose plot shows the wind speed and direction over the measurement site during (**e**) winter, (**f**) pre-monsoon, (**g**) monsoon, and (**h**) post-monsoon seasons. Maximum WS is observed during the pre-monsoon season, and the minimum is noticed during the post-monsoon season. Three days of air-parcel back trajectories are shown at the Eastern Himalayas using the HYSPLIT model at an initial height of 500 m during (**i**) winter, (**j**) pre-monsoon, (**k**) monsoon, and (**l**) post-monsoon seasons. Arrows indicate spatial distribution of wind vectors over the Indian subcontinent region. Back-trajectory analysis shows the movement of wind from the western direction in winter and pre-monsoon seasons, and from the eastern direction in monsoon and post-monsoon seasons.

Seasonal averages of meteorological parameters, (a) temperature, (b) RH, and (c) solar radiation, are shown in Fig. 2. Winter (dry) is characterized by minimum temperature (7 ± 3°C), low solar radiation (122 ± 84 W m^−2^), and high RH (89 ± 7%). The term “dry” refers to negligible rainfall, despite relatively high RH caused by low temperatures and localized air masses. Pre-monsoon (semi-dry) has maximum solar radiation (418 ± 194 W m^−2^) with increased temperatures up to 15 ± 2°C and the lowest RH (87 ± 10%). This transitional season is termed “semi-dry” because the atmosphere begins warming and drying, with stronger winds capable of transporting dust and pollutants. Monsoon (wet) has maximum temperature (17 ± 1°C) and RH (97 ± 3%), with moderate solar radiation (145 ± 114 W m^−2^) due to heavy cloud cover. The label “wet” reflects the dominance of humid air masses from the Bay of Bengal, high precipitation, and persistent cloud coverage. Post-monsoon (semi-wet) is characterized by cooling temperatures (13 ± 4°C) with similar monsoonal solar radiation of about 149 ± 116 W m^−2^ and high RH (91 ± 5%). This season is described as “semi-wet” because, although precipitation declines compared to monsoon, the hilltop site often remains immersed in clouds, maintaining a moist environment favorable for airborne bacterial survival.

PM_2.5_, PM_10_, and AQI data were retrieved from the continuous air quality monitoring system of the West Bengal Pollution Control Board for the nearest station located 2 km away from the sampling site (Darjeeling Police Town Office Complex, Chowk Bazaar), and seasonal average values of PM_2.5_ and PM_10_ are shown in Fig. 2d. The maximum concentration of air pollutants was noticed in pre-monsoon (PM_2.5_ = 59 ± 16 µg·m^-^³, PM_10_ = 115 ± 27 µg·m^-^³), followed by monsoon (46 ± 19 µg·m^-^³, 94 ± 37 µg·m^-^³), post-monsoon (38 ± 14 µg·m^-^³, 74 ± 22 µg·m^-^³), and winter (33 ± 4 µg·m^-^³, 65 ± 15 µg·m^-^³). Wind rose plots are shown in Fig. 2e through h, depicting strong variation in WS and WD in four seasons. Maximum WS (1.2 ± 0.7 m·s^−1^) was noticed in monsoon, coming from the eastern direction, followed by pre-monsoon (1.1 ± 0.7 m·s^−1^) from the western direction, and in post-monsoon (0.7 ± 0.4 m·s^−1^) and winter (0.5 ± 0.4 m·s^−1^) from all directions. It is worth mentioning here that hilltop Himalayan wind in winter and post-monsoon is mainly regional hilly wind with very low speed within 1 m·s^−1^.

Spatial distributions of wind vectors and air-parcel back trajectories over Darjeeling were computed for 72 hours at an initial height of 500 m above ground level to identify the possible sources and traveling paths of airborne bacteria, as shown in Fig. 2i through l. The back trajectories in the winter season are predominantly localized and remain within the hilly regions; however, in the pre-monsoon season, wind over the Eastern Himalayas comes from far distances of western India. In contrast, during the monsoon, back trajectories originate from the BoB and travel via eastern India to the Himalayan hilltop site. During the post-monsoon, back trajectories are significantly reduced and confined within the hilly area and are highly localized. The spatial distribution of wind vectors in Fig. 2i through l, during winter and pre-monsoon seasons, shows similar wind patterns blowing from the west IGP toward the measurement site; however, pre-monsoonal wind has a higher speed. On the other hand, monsoonal and post-monsoonal winds show similar traveling paths from an eastern direction toward Darjeeling but at a relatively higher speed in the monsoon season.

## RESULTS

### Seasonal variation in richness and diversity of airborne bacteria over the Eastern Himalayas

A total of 32,573,925 raw reads and 32,221,412 valid reads were obtained after demultiplexing and quality filtering, followed by assignment of unique operational taxonomic units (OTUs) to reads at 97% similarity. Shannon diversity and Chao1 were estimated to determine alpha-diversity. Details of the sequencing data, diversity index, cell counts, meteorological parameters, and air pollutant concentrations are mentioned in Table 1. Sample details of individual samples with SRA accession numbers are mentioned in Table S1.

**TABLE 1:** Details of airborne bacterial sequencing data with meteorological and air quality parameters in different seasons over the Eastern Himalayas

	Winter (*N* = 25)	Pre-monsoon (*N* = 25)	Monsoon (*N* = 23)	Post-monsoon (*N* = 15)
Raw reads	Min = 173,195 Max = 570,333	Min = 242,553 Max = 629,927	Min = 126,135 Max = 889,182	Min = 149,736 Max = 942,464
Valid reads	Min = 172,901 Max = 544,413	Min = 242,215 Max = 628,866	Min = 125,974 Max = 884,686	Min = 149,497 Max = 940,895
OTUs	239 ± 87	597 ± 343	332 ± 171	492 ± 299
Genera	105 ± 37	188 ± 76	122 ± 58	171 ± 65
Cells × 10^5^ (m^−3^)	3.3 ± 1.1	5.8 ± 1.9	3.4 ± 1.1	4.9 ± 1.5
Shannon diversity	3.4 ± 0.8	4.1 ± 1.0	3.3 ± 1.0	4.1 ± 0.5
Chao1	341 ± 128	659 ± 185	364 ± 132	494 ± 139
Temperature (°C)	7 ± 3	15 ± 2	17 ± 1	13 ± 4
RH (%)	89 ± 7	87 ± 10	97 ± 3	91 ± 5
WS (m·s^−1^)	0.5 ± 0.4	1.1 ± 0.7	1.2 ± 0.7	0.7 ± 0.4
WD (deg.)	205 ± 29	251 ± 36	120 ± 19	179 ± 34
Solar rad. (W·m^−2^)	122 ± 84	418 ± 194	145 ± 114	149 ± 116
PM_2.5_ (µg·m^-^³)	33 ± 4	59 ± 16	46 ± 19	38 ± 14
PM_10_ (µg·m^-^³)	65 ± 15	115 ± 27	94 ± 37	74 ± 22
AQI	67 ± 14	119 ± 30	94 ± 36	79 ± 21

**Fig 3:**
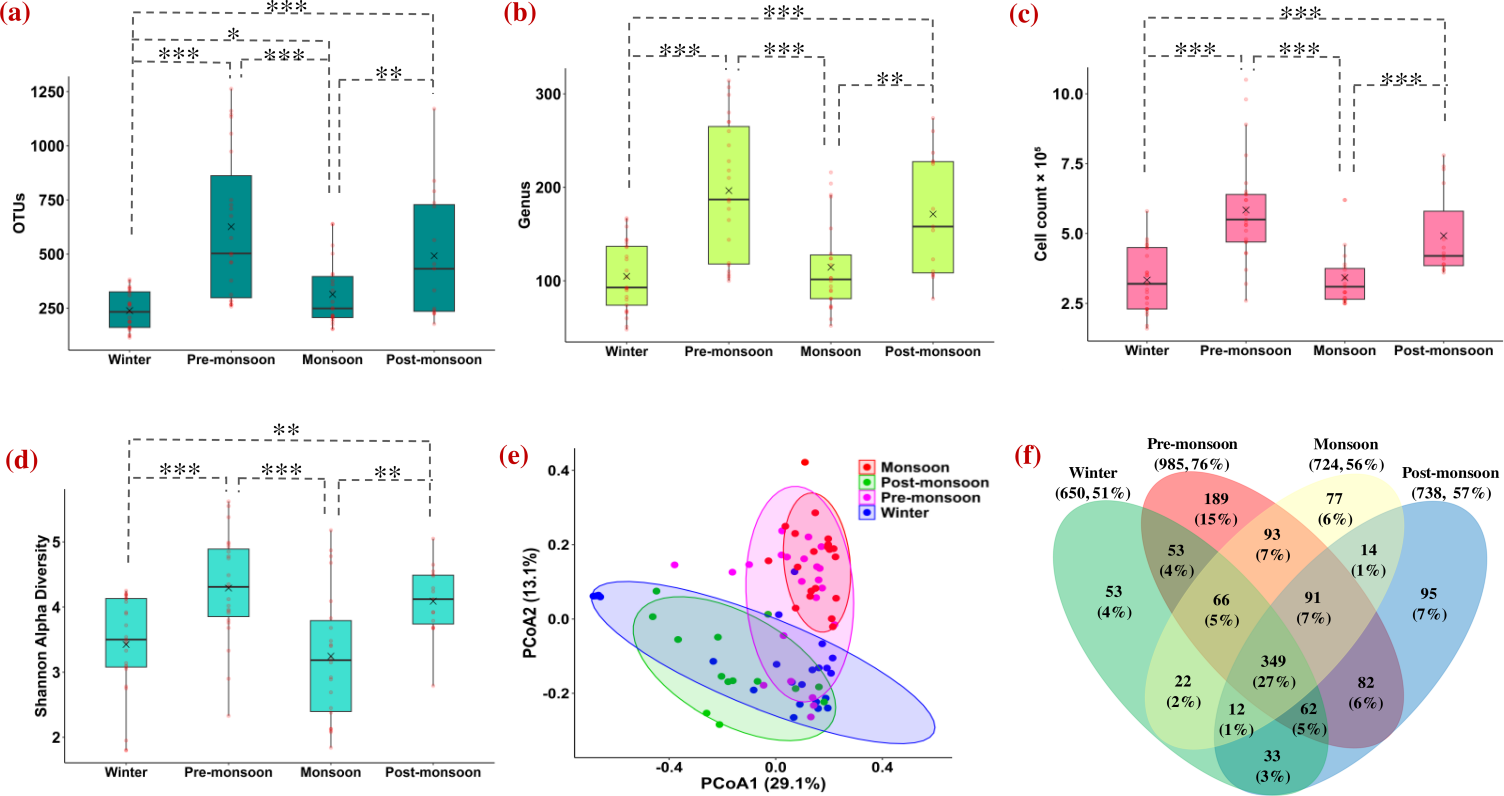
Seasonal variation of (**a**) OTUs, (**b**) genus, (**c**) cell count × 10^5^, and (**d**) Shannon alpha-diversity over the Eastern Himalayas is represented by a box plot, where the maximum concentration of airborne bacterial loading was noticed during pre-monsoon season, followed by post-monsoon, monsoon, and winter seasons. Horizontal lines within boxes represent median, × signifies mean, standard deviations are shown with vertical lines, and red dots represent each sample. (**e**) Beta-diversity is represented as principal coordinate analysis (PCoA) plot during different seasons over the Eastern Himalayas. Shaded regions represent areas of similar beta-diversity, and each point represents an individual sample. (**f**) Venn diagram shows the distribution of common and unique bacterial genera during different seasons. About one-third of the bacterial genera are common to all seasons, and the pre-monsoon season has the highest number of unique bacterial genera (15%). Asterisks indicate significance levels: ***P < 0.001; **P < 0.01; *P < 0.05.

Mean OTUs, genera, cell count, and Shannon diversity during different seasons are shown in Fig. 3a through d. Maximum concentration of airborne bacterial loadings was noticed in pre-monsoon (OTUs = 597 ± 343, genera = 188 ± 76, gells × 10^5^ (m^−3^) = 5.8 ± 1.9, and Shannon diversity = 4.1 ± 1.0), followed by post-monsoon (492 ± 299, 171 ± 65, 4.9 ± 1.5, and 4.1 ± 0.5), monsoon (332 ± 171, 122 ± 58, 3.4 ± 1.1, and 3.3 ± 1.0), and winter (239 ± 87, 105 ± 37, 3.3 ± 1.1, and 3.4 ± 0.8) seasons over the Eastern Himalayas. Beta-diversity was also computed to understand the bacterial diversity in different seasons, as shown in Fig. 3e. Bacterial community composition differed significantly in different seasons, as calculated with PERMANOVA (permutational multivariate analysis of variance) (Bray-Curtis, *r* = 0.17, *F* = 5.36, *P* < 0.001). A Venn diagram (Fig. 3f) indicated that ~25% of bacterial genera were shared across all four seasons, while 36% were common between winter and post-monsoon, and 46% between pre-monsoon and monsoon.

### Effect of meteorological parameters on airborne bacterial communities

**Fig 4:**
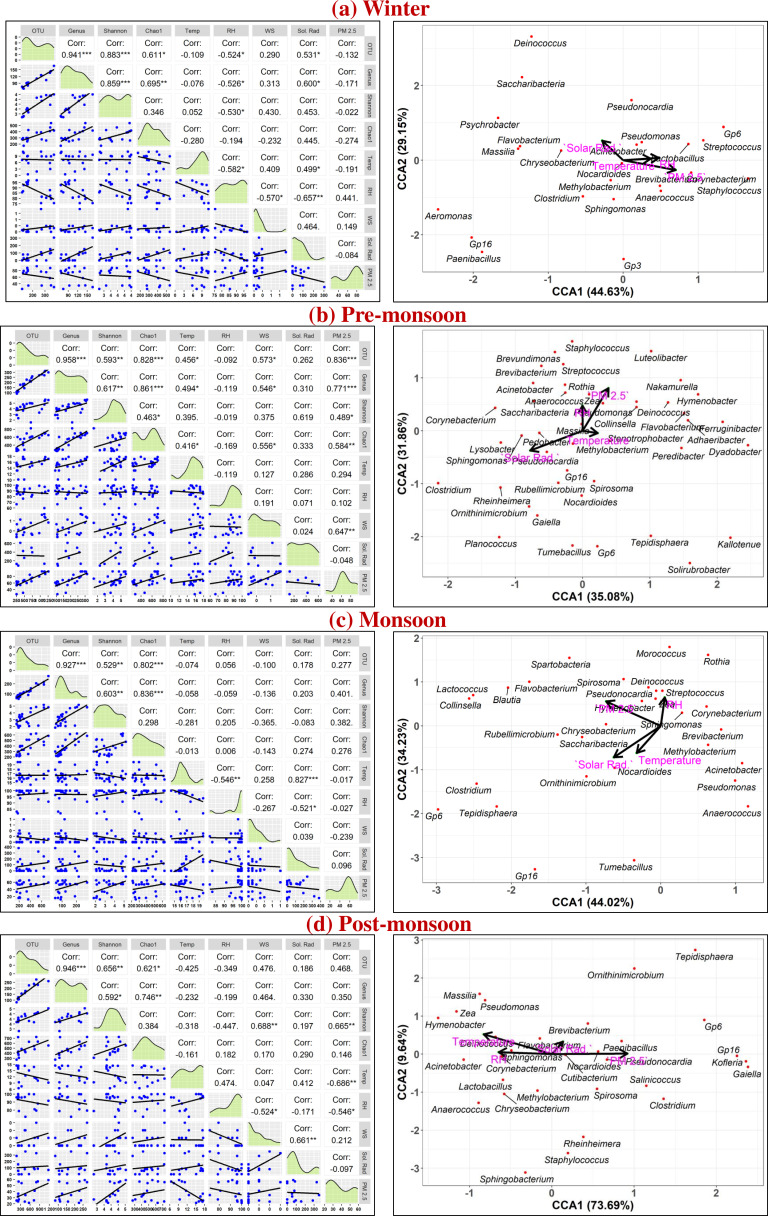
In the right column, Spearman’s correlation is represented by correlation coefficients in the upper triangle. Asterisks indicate significance levels: ***P < 0.001; **P < 0.01; *P < 0.05; and ▪*P* < 0.1. The lower triangle shows a scatter plot with a trendline representing the relationship between each pair of parameters, and the diagonal contains a density plot of each variable during (**a**) winter, (**b**) pre-monsoon, (**c**) monsoon, and (**d**) post-monsoon. In the left column, the CCA plot establishes the relationship between dominant airborne bacteria (relative abundance >0.8%) and meteorological parameters (temperature, RH, solar radiation) and pollutant concentration (PM_2.5_) during (**a**) winter, (**b**) pre-monsoon, (**c**) monsoon, and (**d**) post-monsoon.

Spearman correlation coefficients were calculated to analyze the dependency of airborne bacterial loadings on meteorological parameters and air pollutant concentration during different seasons, shown in the left column of Fig. 4a through d, and the distribution of different dominant types of airborne bacterial genera (relative abundance >1%) with canonical correspondence analysis (CCA) in different seasons over the Eastern Himalayas is shown in the right column of Fig. 4a through d. A significant positive correlation of airborne bacteria in winter was noticed with solar radiation (OTU [*r* = 0.53, *P* < 0.05], genus [*r* = 0.60, *P* < 0.05]), and a negative correlation was observed with RH (OTU [*r* = −0.52, *P* < 0.05], genus [*r* = −0.53, *P* < 0.05], Shannon diversity [*r* = −0.53, *P* < 0.05]). Meteorological dependency of airborne bacterial loading in pre-monsoon is positively correlated with temperature (OTU [*r* = 0.46, *P* < 0.05], genus [*r* = 0.49, *P* < 0.05], and Chao1 [*r* = 0.41, *P* <0.05]), WS (OTU [*r* = 0.57, *P* < 0.05], genus [*r* = 0.55, *P* < 0.05], and Chao1 [*r* = 0.55, *P* < 0.05]), and PM_2.5_ (OTU [*r* = 0.84, *P* < 0.001], genus [*r* = 0.77, *P* < 0.001], Shannon diversity [*r* = 0.49, *P* < 0.05]), and Chao1 [*r* = 0.58, *P* < 0.01]). However, in the monsoon season, significant meteorological dependency of airborne bacterial loadings was not observed due to extreme rainfall and unstable atmospheric conditions. In the post-monsoon season, significant positive correlation of Shannon diversity was noticed with WS (*r* = 0.68, *P* < 0.01) and PM_2.5_ (*r* = 0.66, *P* < 0.01).

CCA plot shows that dominant bacterial genera like *Acinetobacter, Pseudomonas, Chryseobacterium,* and *Pseudonocardia* are positively associated with solar radiation (*F* = 1.46, *P* = 0.10) and negatively related to temperature (*F* = 0.91, *P* = 0.50), RH (*F* = 1.56, *P* = 0.06), and PM_2.5_ (*F* = 1.57, *P* = 0.07), while bacterial genera like *Corynebacterium, Brevibacterium, Lactobacillus, Anaerococcus,* and *Staphylococcus* are positively correlated with temperature, RH, and PM_2.5_ and negatively correlated with solar radiation in winter over the Eastern Himalayas. In pre-monsoon, dominant bacteria like *Deinococcus, Streptococcus, Pseudomonas, Saccharibacteria, Anaerococcus,* and *Massilia* are positively correlated with temperature (*F* = 0.70, *P* = 0.79), RH (*F* = 1.79, *P* = 0.05), and PM_2.5_ (*F* = 1.36, *P* = 0.19) and negatively correlated with solar radiation (*F* = 1.78, *P* = 0.06); however, a few bacteria like *Sphingomonas, Pseudonocardia, Lysobacter,* and *Pedobacter* are positively correlated with solar radiation and negatively correlated with temperature, RH, and PM_2.5_. In monsoon, dominant airborne bacterial genera are equally distributed due to unstable atmospheric conditions and continuous rain. Dominant bacterial genera like *Nocardioides, Paenibacillus, Salinicoccus,* and *Cutibacterium* are positively correlated with PM_2.5_ (*F* = 1.43, *P* = 0.19) and negatively related to RH (*F* = 0.95, *P* = 0.48) and temperature (*F* = 3.36, *P* = 0.01) in the post-monsoon season.

### Dominant sources of airborne bacteria in different seasons over the Eastern Himalayas

**Fig 5:**
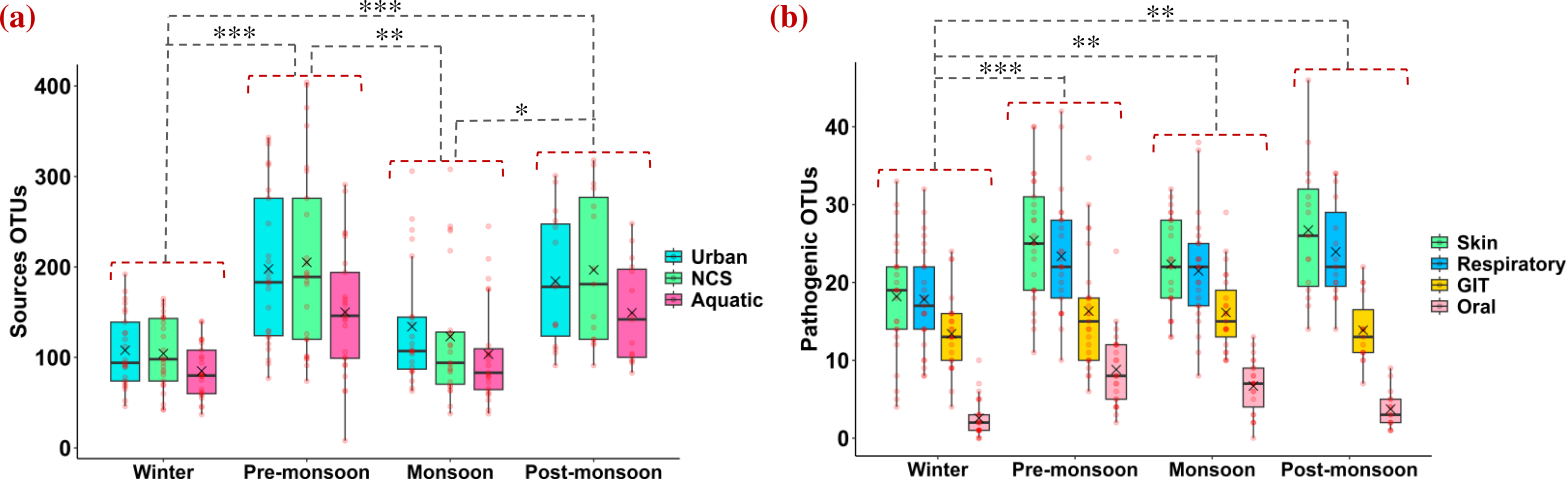
(**a**) Box plot shows seasonal variation in sources of bacterial genera, such as urban, NCS, and aquatic during different seasons over the Eastern Himalayas. (**b**) Mean pathogenic OTUs responsible for skin, respiratory, GIT, and oral infections during different seasons are represented with box plot. Horizontal lines within boxes represent median, × signifies mean, standard deviations are shown with vertical lines, and red dots represent each sample. Asterisks indicate significance levels: ***P < 0.001; **P < 0.01; *P < 0.05.

Airborne bacteria are further categorized according to their sources of isolation, as mentioned in BacDive (Bacterial Diversity Metadatabase) (https://bacdive.dsmz.de/isolation-sources) (35). The identification of sources was conducted using all available strains from a specific genus, demonstrating that most bacterial genera are linked to multiple sources. Sources of bacterial genera are classified into three distinct groups, namely urban sources, which encompass industrial and hospital waste along with other anthropogenic influences; natural continental sources (NCS), which include flora, fauna, agriculture, and soil; and aquatic sources, comprising marine environments, freshwater, and sediments (7). One-way ANOVA (analysis of variance) test shows a significant variation in total sources OTUs from winter to pre-monsoon (*P* < 0.001), from winter to post-monsoon (*P* < 0.001), pre-monsoon to monsoon (*P* < 0.01), and monsoon to post-monsoon (*P* < 0.05) (Fig. 5a). The pre-monsoon season has the highest concentration of all sources (urban = 198 ± 86, NCS = 205 ± 104, aquatic = 150 ± 71), followed by post-monsoon (urban = 184 ± 73, NCS = 197 ± 83, aquatic = 149 ± 56), monsoon (urban = 134 ± 67, NCS = 123 ± 74, aquatic = 103 ± 56), and winter (urban = 108 ± 41, NCS = 104 ± 39, aquatic = 85 ± 30). Both urban and NCS OTU sources are maximum in pre-monsoon, suggesting increased resuspension and transport of terrestrial and anthropogenic particles during this season. Aquatic sources are minimum in all seasons and follow the same seasonal trend, as maximum in pre-monsoon, followed by post-monsoon, monsoon, and winter.

### Pathogenic implications of airborne bacteria on human health in different seasons

A total of 161 pathogenic bacterial genera (winter = 81, pre-monsoon = 95, monsoon = 87, post-monsoon = 86) are present over Darjeeling based on 16S PIP (36). Maximum concentration of pathogenic OTUs was noticed in pre-monsoon (74 ± 25), followed by post-monsoon (68 ± 18), monsoon (67 ± 18), and winter (52 ± 19). The highest concentration of pathogenic OTUs was observed in pre-monsoon due to enhanced long-range transport from Western India, as well as favorable meteorological conditions such as high temperature and WS, with frequent appearances of dust plumes that facilitate the aerosolization of bacterial particles (37–41).

All pathogenic bacterial genera identified at the sampling site are further categorized into four groups according to their target human organs: skin, respiratory, GIT, and oral. Skin (35%) infectious bacteria are present in the maximum concentration over the Eastern Himalayas, followed by respiratory (33%), GIT (23%), and oral (9%) (Fig. 5b). Exposure risk through dermal contact and inhalation may be particularly high for populations residing in the Eastern Himalayan region. Pathogenic OTUs that are harmful to skin and respiratory organs are present most abundantly in post-monsoon (skin = 27 ± 9, respiratory = 24 ± 6), followed by pre-monsoon (25 ± 8, 23 ± 8), monsoon (22 ± 6, 21 ± 7), and winter (18 ± 8, 18 ± 7). However, maximum concentration of pathogenic OTUs for GIT and oral infections is found in pre-monsoon (GIT = 16 ± 8, oral = 9 ± 5), followed by monsoon (16 ± 5, 7 ± 3), post-monsoon (14 ± 4, 4 ± 2), and winter (13 ± 5, 3 ± 2).

**Fig 6:**
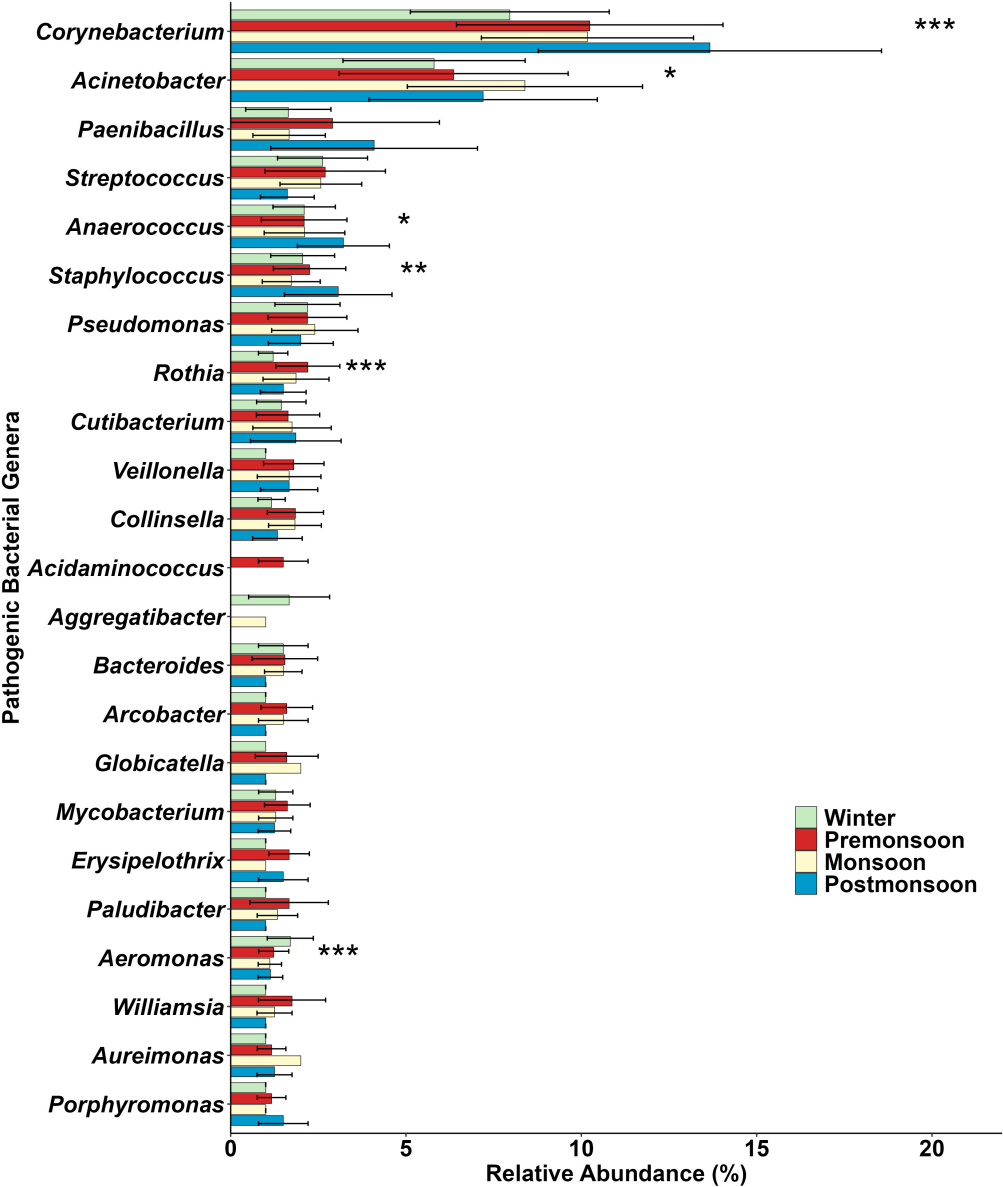
Relative abundance of dominant pathogenic bacterial genera in different seasons over the Eastern Himalayas. Horizontal lines represent standard deviations. Asterisks indicate significance levels: ***P < 0.001; **P < 0.01; *P < 0.05.

Percentages of variation in pathogenic OTUs between different weather conditions are mentioned in Table 2. One-way ANOVA test shows a significant variation in total pathogenic OTUs from winter to pre-monsoon (42%, *P* < 0.001), winter to monsoon (28%, *P* < 0.01), and winter to post-monsoon (31%, *P* < 0.01) as shown in Fig. 5b and mentioned in Table 2. A detailed one-way ANOVA is performed for individual dominant pathogenic genera present in different seasons, and significant results are obtained for *Corynebacterium, Acinetobacter, Anaerococcus, Staphylococcus, Rothia,* and *Aeromonas* (Fig. 6).

**TABLE 2:** Percentage of variation in mean pathogenic OTUs during different seasons^*a*^

Seasons	Skin	Respiratory	GIT	Oral	Total
Winter–Pre-monsoon	40**	31**	22	244***	42***
Winter–Monsoon	23*	20	20*	162***	28**
Winter–Post-monsoon	47**	34*	3	46	31**
Pre-monsoon–Monsoon	14	9	1	31**	11
Pre-monsoon–Post-monsoon	5	2	18	136***	8
Monsoon–Post-monsoon	20	11	16	79**	2

^*a*^ Asterisks indicate significance levels: ***P <0.001; **P <0.01; *P <0.05.

### Role of wind in dispersion of airborne bacteria

**Fig 7:**
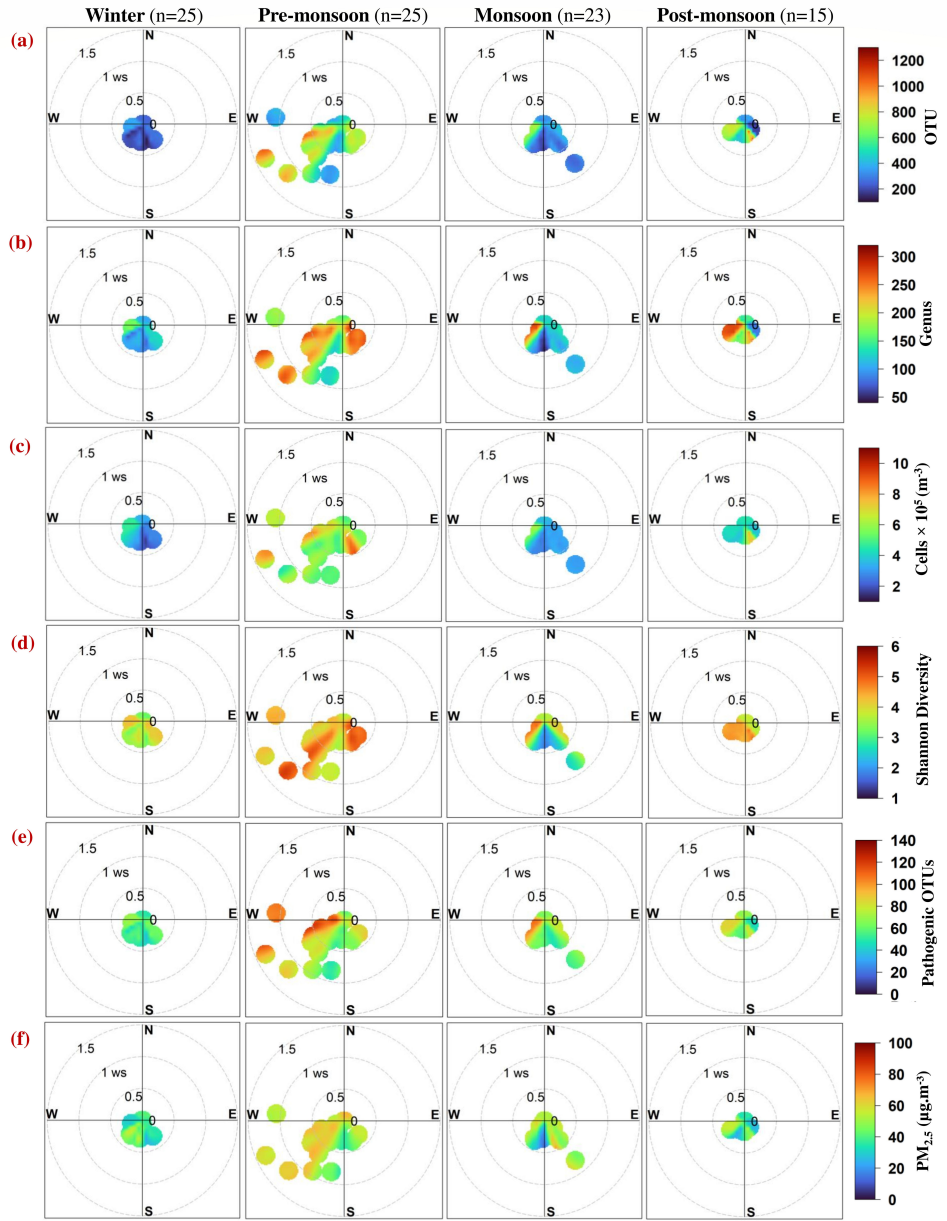
Polar plots show the distribution of (**a**) OTUs, (**b**) genus, (**c**) cell count × 10^5^, (**d**) Shannon diversity, (**e**) pathogenic OTUs, and (**f**) PM_2.5_ along with wind speed (m·s^-1^) and direction during different seasons over the Eastern Himalayas.

Bivariate polar plots illustrate combined variation of a hilltop airborne bacterial species concentration with WS and WD in polar coordinates and provide an effective graphical method for visualizing the directional distribution of atmospheric sources, facilitating the interpretation of source orientation and intensity (42). Bivariate polar plots for (a) OTU, (b) genus, (c) cells × 10^5^ (m^−3^), (d) Shannon diversity, (e) pathogenic OTUs, and (f) PM_2.5_ (µg·m^-^³) in different seasons are shown in Fig. 7. The plots depict the influence of WS and WD on airborne bacterial diversity and pollution levels in the Eastern Himalayas.

Based on prevailing wind conditions during each season, the collected samples are categorized into three groups. The “local” category includes samples associated with calm conditions, defined by zero wind speed, indicating minimal atmospheric transport. The “Eastern India” category comprises samples collected when air masses originated from the eastern part of the country, while the “Western India” category includes those influenced by winds from the western region. This classification enables distinction between locally generated contributions and those associated with regional atmospheric transport. Seasonal influence on the directional contribution (%) of wind from different regions to airborne bacterial communities and pollution parameters over the Eastern Himalayas is mentioned in Table 3.

**TABLE 3:** Seasonal variation in the directional contribution (%) of wind from local, Eastern India, and Western India sources to airborne bacterial communities and pollution parameters

Seasons	Parameters	Local	Eastern India and BoB	Western India and Thar Desert
Winter	OTU	57	6	37
	Genus	55	8	37
	Cells × 10^5^ (m^−3^)	55	5	40
	Shannon diversity	53	8	39
	Pathogenic OTUs	54	7	39
	PM_2.5_ (µg·m^-^³)	53	6	41
Pre-monsoon	OTU	29	10	61
	Genus	31	11	58
	Cells × 10^5^ (m^−3^)	32	10	58
	Shannon diversity	33	10	57
	Pathogenic OTUs	31	8	61
	PM_2.5_ (µg·m^-^³)	39	7	54
Monsoon	OTU	43	19	38
	Genus	45	20	35
	Cells × 10^5^ (m^−3^)	46	20	34
	Shannon diversity	48	20	32
	Pathogenic OTUs	42	20	38
	PM_2.5_ (µg·m^-^³)	49	24	27
Post-monsoon	OTU	38	45	17
	Genus	45	38	17
	Cells × 10^5^ (m^−3^)	48	41	11
	Shannon diversity	49	36	15
	Pathogenic OTUs	53	33	14
	PM_2.5_ (µg·m^-^³)	48	38	14
Annual	OTU	42 ± 12	20 ± 18	38 ± 18
	Genus	44 ± 10	19 ± 14	37 ± 17
	Cells × 10^5^ (m^−3^)	45 ± 10	19 ± 16	36 ± 19
	Shannon diversity	45 ± 9	19 ± 13	36 ± 17
	Pathogenic OTUs	45 ± 11	17 ± 12	38 ± 19
	PM_2.5_ (µg·m^-^³)	47 ± 6	19 ± 15	34 ± 17

Local sources (53%–57%) have the highest contribution, followed by Western India (34%–41%) and Eastern India (5%–8%) in winter, suggesting strong influence of local sources with moderate contribution from Western India. During winter, local sources are dominant, and limited long-range transport is observed under stable atmospheric conditions. While direct evidence for seasonal fluctuation of long-range bacterial transport is limited, previous studies suggest that during colder seasons, stable atmosphere, lower boundary layer heights, and low WS may reduce the influx of distant air masses, thus enhancing the dominance of local sources (3, 43). In the pre-monsoon season, the highest contribution is from Western India (54%–61%), followed by local (29%–39%) and Eastern India (7%–11%), indicating long-range transport of airborne bacteria and pollutants with westerly winds. Previous studies also reported long-range aerosol transport over South Asia (41, 44). The high contribution from Western India during pre-monsoon can be attributed to arid and semi-arid regions of Western India, which act as major dust sources and transport mineral dust attached to bacterial communities toward our sampling site (45). Eastern India (19%–24%) contribution is increased during monsoon, likely due to wind back trajectories from BoB, in addition to local (42%–49%) and Western India (27%–38%) contributions. The enhanced contribution from Eastern India during the monsoon season depicts the influence of regional monsoonal circulation and rainfall, as it can suppress local resuspension and introduce new bacterial populations from marine or coastal environments into the atmosphere (2). Post-monsoon also shows prevailing wind back trajectories from BoB and has high Eastern India contributions (33%–45%), along with local (38%–53%) and Western India (11%–17%), similar to the monsoon season.

It is worth mentioning that PM_2.5_ and pathogenic OTUs follow similar WD trends, indicating a positive relationship between pollutants and pathogenic bacteria. Wind plays a significant role in the dispersion and short- and long-range transport of airborne bacteria. Wind can either enhance or reduce the concentration of airborne bacteria depending on its direction, speed, and sampling site characteristics (46). The Eastern Himalayas provide a unique environment where intricate topography and seasonal monsoonal patterns influence wind-driven bacterial movement. These results emphasize the importance of regional wind patterns in transporting airborne bacteria, as they have significant implications for seasonal exposure risks and public health management over the Eastern Himalayas.

## DISCUSSION

### Environmental factors influencing airborne bacterial abundances

Maximum airborne bacterial loadings are observed in the pre-monsoon season compared to other seasons, primarily due to the accumulation of airborne bacteria in this season over hilltop Himalayas. Previous studies have also reported the enhancement of airborne bacterial loadings due to increased solar radiation, WS, and long-range transport (47, 48). In contrast, minimum concentration is noticed in winter, as the temperature is very low (7 ± 3°C) with the lowest concentration of air pollutants (PM_2.5_ =33 ± 4 µg·m^-^³, PM_10_ = 65 ± 15 µg·m^-^³), which are generally unfavorable for bacterial survival (49). Additionally, low solar radiation (122 ± 84 W·m^−2^) and reduced convective activity may restrict vertical mixing and long-range transport of airborne bacteria during winter. Airborne bacterial populations present in winter are considered as the background atmosphere, as the boundary layer height is very low and the free troposphere is evident over the measurement site.

In monsoon season, there is a reduction of airborne bacterial loadings as the wind is blown from the east direction via BoB, and, due to rain, washout of airborne bacterial loadings occurs from the atmosphere (50). Another study over the Tibetan Plateau and nearby glaciers has reported that the Indian monsoon can increase airborne bacterial diversity at high elevations through advection of humid, biologically rich air masses, including dust-associated taxa from desert sources during monsoon transport events (51, 52). This difference is likely due to altitude and source-proximity effects: the Tibetan glacier sites receive long-range monsoonal inflow that augments diversity aloft, whereas our hilltop site experiences stronger wet deposition that can suppress airborne bacterial loadings (51). However, during the post-monsoon season, enhancement of airborne bacterial loadings is observed because of the presence of dense clouds and foggy conditions, which possibly favor proliferation of airborne bacteria by providing nutrients and water vapor for their growth (7, 53). Similar seasonal variations are also observed in arid and temperate regions, where airborne bacterial loadings are often highest during transitional weather conditions (54, 55).

Spearman’s correlation further explains the role of meteorological parameters and air pollution on airborne bacterial loadings. A positive correlation with solar radiation in winter indicates that higher solar radiation may assist in aerosolization and dispersal of soil- and surface-bound bacteria, thereby increasing their abundance in the atmosphere (56). In contrast, an increase in RH promotes aggregation of airborne particles, which facilitates deposition from the atmosphere, and therefore, a negative correlation is noticed with airborne bacterial loadings. Similar results of negative correlations with RH are also reported over the USA, Tokyo, and Greenland (37, 48, 50). In pre-monsoon, positive correlations with temperature, WS, and PM_2.5_ highlight the combined effects of both favorable meteorological conditions and pollutant concentrations on airborne bacterial communities, which are associated with long-range air mass transport from the continental regions (29, 47, 57). Ambient bacterial loadings also correlate positively with temperature during pre-monsoon in different Indian cities like Dehradun, located in Doon Valley on the foothills of Himalayas (58), and Bangalore, an urban metropolitan city (59). PM_2.5_ shows a strong positive correlation with OTUs, indicating its role as a vector for airborne bacterial dispersion over the Eastern Himalayas during the pre-monsoon season (38, 56, 60). During intense cloudy conditions in the post-monsoon, PM_2.5_ helps in the sustenance of the airborne bacterial population in the atmosphere. The interplay of meteorological parameters, pollutant concentrations, and atmospheric transport processes strongly regulates seasonal differences in bacterial loadings over the Eastern Himalayas.

### Influence of air-mass sources on airborne bacterial communities

Air mass plays a significant role in the alteration of airborne bacterial communities. In the principal coordinate analysis (PCoA) plot, ellipses are formed on the basis of Bray-Curtis dissimilarity distances, which help to cluster the sampling points, as shown in Fig. 3e. Four distinct ellipses are formed, representing winter, pre-monsoon, monsoon, and post-monsoon seasons. However, there is an overlap between winter with post-monsoon season and pre-monsoon with monsoon season, indicating transitional similarities driven by similar wind flow and environmental conditions during these two seasons. A previous study also reported such overlap in airborne bacterial communities across winter, spring, and summer over an urban atmosphere (61). At a high-altitude site of Mount Sonnblick Observatory (3,106 m amsl) in Austria, there is a strong overlap in the airborne bacterial samples collected in May and August, and samples from February have the most distinct airborne bacterial population (62).

During winter and post-monsoon, wind is mostly localized, which governs the diversity of airborne bacteria, and, as a result, similar bacterial compositions (about 36%) are noticed during these seasons (Fig. 3f). Similarly, pre-monsoon and monsoon seasons have overlapping beta-diversities, and about 46% of bacterial genera are common between these seasons, as shown in the Venn diagram (Fig. 3f). Overall, about one-fourth of airborne bacterial genera are common in all four seasons and are considered the background atmosphere of the Eastern Himalayas. This consistent airborne bacterial community is influenced by local soils, vegetation, and human or animal activity and represents a stable background for this region. Seasonal clustering and overlaps demonstrate that both localized winds and long-range air-mass transport regulate airborne bacterial community structures in the Eastern Himalayas.

### Season-specific bacterial taxa and pathogenic implications

Seasonal dominance of specific pathogenic taxa has important health implications. *Corynebacterium* (13.7 ± 4.9%) is most abundant in post-monsoon and can cause diphtheria, pharyngitis, fever, respiratory tract infections, urinary tract infections (UTIs), wound infections, and endocarditis in humans (63). *Corynebacterium* is also infectious to cattle and causes mastitis and abscesses (64). *Acinetobacter* is present in the highest concentration during monsoon (8.4 ± 3.4%) and is responsible for nosocomial infections like bacteremia and UTIs (65). *Anaerococcus* is also abundant in post-monsoon (3.2 ± 1.3%) and is infectious for skin and soft tissues (66). *Staphylococcus* is abundant in post-monsoon (3.1 ± 1.5%) and can cause skin lesions, osteomyelitis, endocarditis, furunculosis, food poisoning, and UTIs in humans (67). *Staphylococcus* is also highly infectious in animals and causes pyoderma, exudative dermatitis, endocarditis, cutaneous granuloma, botryomycosis, pyogenic meningoencephalitis, skin lesions, and sepsis, and nasal carriage (68). *Rothia* is present in the highest concentration in pre-monsoon (2.2 ± 0.9%) and is responsible for bacteremia, central nervous system infection, meningitis, peritonitis, osteomyelitis, cervical necrotizing fasciitis, endophthalmitis, and endocarditis in humans (69). *Aeromonas* is abundant in winter (1.7 ± 0.7%) and is responsible for a variety of extraintestinal and systemic infections, as well as gastrointestinal infections in humans (70). *Aeromonas* is also pathogenic to poikilothermic animals and associated with diseases of birds and mammals (70). The presence of these pathogenic airborne bacterial genera over the Eastern Himalayas throughout the year poses significant health implications for the people residing in the hilltop atmosphere.

Several unique pathogenic bacterial genera are identified across different seasons, indicating distinct seasonal alterations of airborne pathogens. During the pre-monsoon season, multiple pathogens are exclusively detected, including *Erysipelothrix* (2.0 ± 0.1%), which is known to cause cutaneous lesions (71); *Dysgonomonas* (1.3 ± 0.6%), which is associated with bacteremia and leukemia in immunocompromised individuals (72); *Peptostreptococcus* (1.3 ± 0.6%), which is implicated in UTIs (73); *Sarcina* (1.3 ± 0.5%), which causes chronic nausea, dyspepsia, and abdominal discomfort (74); *Campylobacter* (1.3 ± 0.5%), which is responsible for diarrhea (75); and *Thermoactinomyces* (1.1 ± 0.3%), which causes lower respiratory tract infections typically acquired through inhalation of dust particles (76). These pathogens are not detected in other seasons and are hypothesized to have been transported from the western IGP via seasonal air-mass movements. However, a few pathogens are uniquely introduced from the eastern direction in monsoon, like *Globicatella* (2.0 ± 0.5%), which causes bacteremia (77), and *Selenomonas* (1.2 ± 0.5%), the primary pathogen in tooth decay (78). Localized wind in post-monsoon and winter seasons has a regional wind pattern influence on the presence of unique pathogens like *Cellulomonas* (1.3 ± 0.6%), which causes bacteraemia and sepsis (79); *Aggregatibacter* (1.7 ± 1.2%), a periodontal pathogen in the oral cavity (80); and *Lawsonella* (1.1 ± 0.3%), which causes abdominal, breast, and spinal abscesses (81). These findings highlight that meteorological parameters and transport pathways shape bacterial diversity and govern the seasonal prevalence of clinically relevant pathogens, underscoring potential risks for both human and animal health in the Eastern Himalayas.

### Conclusions

To study the role of wind in the alteration of Eastern Himalayan bacterial diversity and pathogenicity, air samples were collected over Darjeeling (27.03°N, 88.26°E, 2,200 m amsl) from January 2022 to September 2023. Significant seasonal shifts were observed in Himalayan airborne bacterial composition, with maximum loading and richness in pre-monsoon, followed by post-monsoon, monsoon, and winter. Airborne bacterial loadings had a strong positive correlation with wind speed (*r* = 0.57, *P* < 0.05), temperature (*r* = 0.50, *P* < 0.05), and PM_2.5_ (*r* = 0.84, *P* < 0.001), indicating the governing role of meteorological parameters. One-fourth (349) of airborne bacterial genera are independent of seasonal changes, implying a background of the Eastern Himalayas. Maximum unique airborne bacterial genera are present during pre-monsoon (15%), followed by post-monsoon (7%), monsoon (6%), and winter (4%) seasons. Air-parcel back-trajectory analysis suggests that pre-monsoon communities are enriched with continental airborne bacteria transported from western IGP, whereas monsoon winds carry marine bacteria from BoB. In winter and post-monsoon season, airborne bacteria are mostly localized. The present study highlights the significant influence of atmospheric transport processes on the alteration of Eastern Himalayan airborne bacterial population. Human pathogens like *Aeromonas, Acinetobacter, Staphylococcus*, and *Corynebacterium,* causing gastroenteritis, endocarditis, respiratory, skin, and urinary tract infections, are dominating throughout the year over the Eastern Himalayas. However, unique pathogens such as *Erysipelothrix* (cutaneous lesions), *Dysgonomonas* (bacteraemia and leukemia), *Peptostreptococcus* (UTIs), *Sarcina* (gastrointestinal symptoms), *Campylobacter* (diarrhea), and *Thermoactinomyces* (respiratory infections) are transported from western IGP in premonsoon season, while winds from BoB carried a few unique pathogens like *Globicatella* (bacteraemia) and *Selenomonas* (tooth decay) in monsoon. Post-monsoon and winter seasons have regional hilly winds, and some unique pathogens are detected in these seasons, like *Cellulomonas* (bacteraemia and sepsis), *Aggregatibacter* (periodontal disease), and *Lawsonella* (abscesses). The dynamic interplay between meteorological conditions, air quality parameters, and airborne bacterial diversity demands integrated monitoring strategies that include atmospheric biological data and meteorological modeling, which are essential for public health in the Eastern Himalayan regions.

## MATERIALS AND METHODS

### Sample collection

Airborne samples are collected from the Bose Institute campus, Darjeeling, from January 2022 to September 2023. Samples are collected for 8 hours, three times a day, at a height of 15 m above ground, and 88 samples are collected in total. It is worth mentioning here that the height of the inlet is above the local canopy. Ambient air is passed through sterile 47 mm radium filter paper of 0.22 µm pore size (Mixed Cellulose Ester Membrane, Merck Life Science Pvt. Ltd., India) continuously using air pumps. Details of the sample collection protocol are mentioned in the literature (7, 53), and a brief description is mentioned here for the sake of completeness. After sample collection, filters are grouped into two parts. One group is transferred to TE buffer (50 mM Tris, 50 mM EDTA, [pH 7.8]) for metagenomic study and stored at −20°C in the laboratory. The second group is transferred to NSG buffer (normal saline glycerol, 15% glycerol, 0.9% NaCl solution) for total cell count study and stored at 4°C. Samples are stored in a cryo-box, and then the cryo-box is kept in an ice box to maintain a temperature of 4°C during transportation.

### DNA isolation and total cell count quantification

Microbial cell suspension is prepared according to the protocol mentioned in the literature (7). The DNeasy PowerWater Kit (Qiagen) is used according to the manufacturer’s protocol to extract DNA from microbial cell suspension prepared from ambient air samples stored in TE buffer. The concentration of extracted DNA is measured using Qubit DNA HS Assay (Invitrogen). As air samples yield very low concentrations of DNA, contamination from additional sources might alter the microbiome analysis (7, 82, 83). Therefore, blank samples and kitome (isolated from the Qiagen DNeasy PowerWater Kit) are also processed simultaneously, and the reads obtained from them are eliminated from air samples collected during the sample collection period.

Microbial cell suspension prepared from filters stored in NSG buffer is used for total cell count study. The cells are stained with 2 µL of 10 mM 4′,6-diamidino-2-phenylindole (Thermo Scientific, USA), and the number of cells is determined using phase contrast and fluorescence microscopy on a hemocytometer (60, 84, 85). A detailed protocol for total cell count is mentioned in the literature (7).

### Amplification of V3-V4 regions of 16S rRNA gene fragments and sequencing by Illumina NovaSeq 6000

The V3-V4 regions of 16S rRNA genes are amplified by polymerase chain reaction (PCR) using the “Fusion primer protocol” for bacterium-specific universal oligonucleotides. PCR is performed on DNA samples using the universal forward primer, 341f (5′-CC TAC GGG NGG CWG CAG-3′), and the universal reverse primer, 805r (5′- GAC TAC HVG GGT ATC TAA TCC-3′). PCR reaction is carried out using Platinum Taq DNA Polymerase, High Fidelity (Invitrogen, Thermo Fisher Scientific) for 35 cycles of 94°C for 30 seconds, 55°C for 30 seconds, and 68°C for 1 minute. After amplification, all PCR products are purified with AMPure XP beads according to the manufacturer’s protocol. Purified PCR products are again amplified with Index PCR—Index 1 and 2 primers available in Nextera XT Index Kit v.2 set (Illumina) for 35 cycles, as mentioned above. After amplification with index primers, PCR clean-up 2 is performed with AMPure XP beads, and quantification of the final library is measured with Qubit dsDNA HS Assay Kit (Invitrogen, Thermo Fisher Scientific). Multiple samples are then pooled in equal concentrations for Illumina NovaSeq 6000 sequencing with Illumina reagent kits according to the manufacturer’s protocol, which results in an average read length of >350 bp.

### Taxonomic classification and diversity profiling of airborne bacteria

To eliminate DNA contamination, 100% aligned reads from kitome and blank samples are excluded from air sample reads with the short-read aligner Bowtie (v.2.4.4) in default stagnant with end-to-end and very sensitive alignment mode. A fast and accurate Illumina Paired-End reAd meRger (PEAR v.0.9.10) is used to merge paired-end reads after elimination of sequences from blanks and kitome (86). After the elimination of DNA contamination, the sequence reads of these extracted sequences are again filtered for a quality value of 20 and a sequence length threshold of 100 bp with Fastx-toolkit (v.0.0.13). After that, using modules from UPARSE, OTUs are clustered at 97% similarity; singletons are also discarded while clustering OTUs (87). The RDP classifier is used at an 80% confidence level to determine the taxonomic affiliation of sequences of each OTU. Shannon diversity and Chao1 are determined using a Perl programming script available within UPARSE. A high-throughput sequencing shows that plateaus are obtained in rarefaction curves, indicating that dominant phylotypes are captured in each sample.

Taxonomic profiling is performed in the Galaxy platform (88). Quality check of 16S amplicon data is performed with FastQC (version 0.12.1) (89) and trimmed with Trim Galore! (version 0.6.7) (90). Kraken2 (version 2.1.3) is used to perform taxonomic profiling with Silva (Created: 2022-02-02T162959Z, kmer-len = 35, minimizer-len = 31, minimizer-spaces = 6, load-factor = 0.7) as the reference database to map the reads (91). The confidence score in Kraken2 is set to 10% to classify maximum reads. Report files from Kraken2 and metadata files are then combined with Kraken-biom (version 1.2.0) to form a biom format file (92). In R programming language, the phyloseq (version 1.48.0) package is used to calculate beta-diversity (93). The Plot_ordinate function is used to calculate PCoA values and Bray-Curtis distances for beta-diversity analysis.

### Identification of sources and pathogenicity of airborne bacterial genera

All bacterial genera are subsequently investigated to determine their sources using BacDive (the Bacterial Diversity Metadatabase) (35). Source identification is carried out using accessible strains from a certain genus, and most bacterial genera are noticed to have several sources. Based on the database, sources of bacterial genera are grouped into three categories: natural continental (flora, fauna, agriculture, soil), urban (industrial and hospital waste, other anthropogenic sources), and aquatic (marine, freshwater, sediments).

Pathogenic airborne bacteria are detected using a pipeline for identification of pathogens in fast mode based on 16S metagenomic sequences (16S PIP) (36). Based on their target organs in humans, these pathogenic airborne bacteria are classified into four groups: skin, respiratory, GIT, and oral (53). Pathogenic genera that target these organs are evaluated because they come into direct contact with air. Other pathogenic genera that cause various diseases, such as bacteremia, septicemia, meningitis, liver infections, endocarditis, etc., are not considered in the current study, as humans have no direct air contact with these internal illnesses.

### Computation of back trajectories and wind vectors

The Hybrid Single-Particle Lagrangian Integrated Trajectory (HYSPLIT) model is used for computation of air-parcel back trajectories over Darjeeling to investigate possible sources and traveling paths of the air parcel. Three days of back trajectories at an initial height of 500 m above ground level over the measuring site are computed during different seasons and shown in Fig. 2i through l. The spatial distribution of wind vectors (at 995 mb) over the Indian subcontinental region is obtained from National Centers for Environmental Prediction–National Center for Atmospheric Research (NCEP-NCAR) reanalysis data and demonstrated during different seasons in Fig. 2i through l.

### Statistical analysis

Spearman correlation analysis between airborne bacterial loadings and meteorological parameters such as temperature, RH, WS, WD, solar radiation, and PM_2.5_ is obtained using corrplot (version 0.92) and GGally (version 2.2.1) packages in the R programming language, and *P*-values of <0.05 are statistically significant. CCA is performed with the cca function of the vegan (version 2.6-6.1) package, and polar plots are obtained using the polarPlot function of the openair package (version 2.18.2.9008) in the R programming language. PERMANOVA is performed with the adonis2 function of the vegan (version 2.6-6.1) package in the R programming language. The 95% confidence interval for the meteorological and air pollution data is computed using the t.test function in R. One-way ANOVA is performed for all four seasons with PAST software (version 4.03).

## Data Availability

The sequence files are deposited in the Sequence Read Archive (SRA) of National Center for Biotechnology Information (NCBI), USA, under the BioProject accession number PRJNA1271967. PM_2.5_, PM_10_, and AQI data were retrieved from continuous air quality monitoring system of West Bengal Pollution Control Board (https://www.wbpcb.gov.in/). The shapefile used to plot state boundaries in Fig. 1a and district boundaries in Fig. 1b of India was retrieved from Administrative Boundary Database, Survey of India, Ministry of Science and Technology (https://onlinemaps.surveyofindia.gov.in/), and plotted with the help of QGIS (3.40.0). 100 m resolution Population Count of India 2020 was retrieved from WorldPop (https://hub.worldpop.org/doi/10.5258/SOTON/WP00645). India’s elevation profile in GeoTIFF file was retrieved from Figshare (https://figshare.com/articles/dataset/India_s_elevation_profile_in_a_GeoTIFF_file/12479306).

## SUPPLEMENTAL MATERIAL

Table S1 aem.02187-25-s0001.docx Details of sample collected from Eastern Himalayas with sequencing data, meteorological parameters, and SRA accession numbers.

ASM does not own the copyrights to Supplemental Material that may be linked to, or accessed through, an article. The authors have granted ASM a non-exclusive, world-wide license to publish the Supplemental Material files. Please contact the corresponding author directly for reuse.
