# Supplementary material for: Role of wind in alteration of hilltop airborne bacterial communities enriched with pathogens over the Eastern Himalayas in India
Source: Appl Environ Microbiol. 2025 Dec 18;92(1):e02187-25. doi: 10.1128/aem.02187-25 (PMC12838236; doi:10.1128/aem.02187-25)
Supplement: Table S1 — Details of sample collected from Eastern Himalayas with sequencing data, meteorological parameters, and SRA accession numbers. [file aem.02187-25-s0001.docx]

**Table S1-** Details of sample collected from Eastern Himalayas with sequencing data, meteorological parameters and SRA accession numbers.

**Supplementary Information**

| **Sample ID** | **Seasons** | **Sampling Date**  **(mm/dd/yy)** | **Sampling Time** | **Sample Duration** | **Air Volume (L)** | **Raw reads** | **Valid reads** | **OTU** | **Genus** | **Cells × 10⁵**  **(m^-3^)** | **Shannon Diversity** | **Chao1** | **SRA Accession Numbers** |
| --- | --- | --- | --- | --- | --- | --- | --- | --- | --- | --- | --- | --- | --- |
| D1 | Winter (n=25) | 01-06-22 | 22:00-06:00 | 8h | 11040 | 203470 | 203276 | 207 | 67 | 4.5 | 3.05 | 83 | SRR33854618 |
| D2 |  | 01-07-22 | 06:00-14:00 | 8h | 11040 | 206780 | 206611 | 188 | 51 | 2.3 | 1.95 | 61 | SRR33854617 |
| D3 |  | 01-07-22 | 14:00-22:00 | 8h | 11040 | 231622 | 231421 | 261 | 137 | 3.6 | 2.75 | 103 | SRR33854577 |
| D4 |  | 01-08-22 | 06:00-14:00 | 8h | 11040 | 221739 | 221564 | 269 | 92 | 5.8 | 2.78 | 110 | SRR33854566 |
| D5 |  | 01-08-22 | 14:00-22:00 | 8h | 11040 | 211042 | 210870 | 185 | 48 | 4.8 | 1.8 | 54 | SRR33854555 |
| D6 |  | 01-08-22 | 22:00-06:00 | 8h | 11040 | 237727 | 237523 | 188 | 60 | 2.3 | 1.8 | 60 | SRR33854544 |
| D7 |  | 01-09-22 | 06:00-14:00 | 8h | 11040 | 570333 | 269902 | 325 | 144 | 3.2 | 3.9 | 182 | SRR33854533 |
| D8 |  | 01-09-22 | 14:00-22:00 | 8h | 11040 | 430460 | 430173 | 382 | 165 | 2.3 | 4.13 | 228 | SRR33854522 |
| D9 |  | 01-09-22 | 22:00-06:00 | 8h | 11040 | 544765 | 544413 | 330 | 158 | 2.5 | 4.17 | 201 | SRR33854604 |
| D63 |  | 12-06-22 | 22:00-06:00 | 8h | 11040 | 262124 | 261691 | 164 | 93 | 1.7 | 3.33 | 111 | SRR33854530 |
| D64 |  | 12-08-22 | 06:00-14:00 | 8h | 11040 | 408322 | 407465 | 374 | 167 | 4.7 | 4.25 | 223 | SRR33854529 |
| D65 |  | 12-08-22 | 14:00-22:00 | 8h | 11040 | 407864 | 407175 | 274 | 123 | 3.5 | 3.92 | 158 | SRR33854528 |
| D66 |  | 12-08-22 | 22:00-06:00 | 8h | 11040 | 517042 | 516191 | 114 | 74 | 1.6 | 3.08 | 88 | SRR33854527 |
| D67 |  | 12-09-22 | 14:00-22:00 | 8h | 11040 | 322194 | 321646 | 349 | 144 | 4.6 | 4.19 | 180 | SRR33854526 |
| D68 |  | 01-13-23 | 14:00-22:00 | 8h | 11040 | 343723 | 342938 | 340 | 136 | 4.2 | 4.19 | 190 | SRR33854525 |
| D69 |  | 01-15-23 | 22:00-06:00 | 8h | 11040 | 335253 | 334722 | 233 | 111 | 3.6 | 4.08 | 145 | SRR33854524 |
| D70 |  | 01-17-23 | 14:00-22:00 | 8h | 11040 | 509637 | 508755 | 343 | 141 | 4.5 | 3.72 | 184 | SRR33854523 |
| D71 |  | 01-17-23 | 22:00-06:00 | 8h | 11040 | 448147 | 447413 | 161 | 80 | 2.7 | 3.44 | 98 | SRR33854614 |
| D72 |  | 02-19-23 | 06:00-14:00 | 8h | 11040 | 291978 | 291477 | 121 | 68 | 3 | 3.1 | 90 | SRR33854613 |
| D73 |  | 02-19-23 | 14:00-22:00 | 8h | 11040 | 506962 | 506089 | 127 | 66 | 4.6 | 3.14 | 87 | SRR33854612 |
| D74 |  | 02-19-23 | 22:00-06:00 | 8h | 11040 | 173195 | 172901 | 310 | 104 | 2.9 | 4.21 | 163 | SRR33854611 |
| D75 |  | 02-20-23 | 06:00-14:00 | 8h | 11040 | 375848 | 375226 | 159 | 83 | 2.1 | 3.5 | 111 | SRR33854610 |
| D76 |  | 02-20-23 | 14:00-22:00 | 8h | 11040 | 306975 | 306521 | 155 | 92 | 3.5 | 3.54 | 116 | SRR33854609 |
| D77 |  | 02-20-23 | 22:00-06:00 | 8h | 11040 | 339510 | 338979 | 149 | 90 | 2.2 | 3.47 | 103 | SRR33854608 |
| D78 |  | 02-23-23 | 14:00-22:00 | 8h | 11040 | 472421 | 471655 | 268 | 126 | 2.7 | 4.13 | 164 | SRR33854607 |
| D10 | Pre-monsoon (n=25) | 04-08-22 | 06:00-14:00 | 8h | 11040 | 439972 | 439392 | 1056 | 280 | 7.8 | 3.94 | 325 | SRR33854593 |
| D11 |  | 04-08-22 | 22:00-06:00 | 8h | 8280 | 321352 | 320917 | 258 | 103 | 3.7 | 3.76 | 131 | SRR33854616 |
| D12 |  | 04-10-22 | 14:00-22:00 | 6h 30m | 8970 | 271367 | 271008 | 676 | 218 | 5.4 | 4.99 | 236 | SRR33854615 |
| D14 |  | 04-11-22 | 06:00-14:00 | 8h | 11040 | 386352 | 385854 | 268 | 91 | 5.5 | 2.9 | 84 | SRR33854584 |
| D15 |  | 04-11-22 | 14:00-22:00 | 8h | 11040 | 431246 | 430730 | 709 | 210 | 4.3 | 1.58 | 210 | SRR33854583 |
| D16 |  | 04-11-22 | 22:00-06:00 | 8h | 11040 | 319966 | 319615 | 281 | 117 | 6.2 | 3.91 | 120 | SRR33854582 |
| D17 |  | 05-12-22 | 14:00-22:00 | 8h | 11040 | 301652 | 301150 | 574 | 228 | 4.7 | 4.85 | 237 | SRR33854581 |
| D18 |  | 05-14-22 | 14:00-22:00 | 8h | 11040 | 312727 | 312305 | 285 | 119 | 3.2 | 3.96 | 126 | SRR33854580 |
| D19 |  | 05-14-22 | 22:00-06:00 | 7h 12m | 9936 | 310148 | 309849 | 267 | 100 | 5.3 | 4.12 | 105 | SRR33854579 |
| D20 |  | 05-15-22 | 06:00-14:00 | 8h | 11040 | 342169 | 341698 | 1144 | 307 | 10.5 | 5.38 | 370 | SRR33854578 |
| D21 |  | 05-15-22 | 14:00-22:00 | 7h 30m | 10350 | 323282 | 322861 | 503 | 144 | 5.9 | 4.49 | 163 | SRR33854576 |
| D22 |  | 05-15-22 | 22:00-06:00 | 8h | 11040 | 323446 | 323050 | 258 | 105 | 6.5 | 3.8 | 125 | SRR33854575 |
| D23 |  | 05-16-22 | 06:00-14:00 | 7h | 9660 | 462812 | 462154 | 974 | 270 | 6.2 | 3.33 | 312 | SRR33854574 |
| D24 |  | 05-17-22 | 06:00-14:00 | 8h | 11040 | 337187 | 336748 | 1161 | 314 | 6.4 | 5.62 | 415 | SRR33854573 |
| D25 |  | 05-17-22 | 14:00-22:00 | 8h | 11040 | 242553 | 242215 | 1263 | 299 | 4.8 | 5.55 | 360 | SRR33854572 |
| D26 |  | 05-17-22 | 22:00-06:00 | 8h | 11040 | 308455 | 308051 | 266 | 100 | 5.1 | 4.02 | 120 | SRR33854571 |
| D27 |  | 05-18-22 | 06:00-14:00 | 8h | 11040 | 433282 | 432762 | 462 | 185 | 2.6 | 4.78 | 219 | SRR33854570 |
| D82 |  | 03-16-23 | 14:00-22:00 | 8h | 11040 | 629927 | 628866 | 312 | 110 | 4.7 | 2.33 | 142 | SRR33854602 |
| D84 |  | 03-17-23 | 06:00-14:00 | 7h 30m | 10074 | 548406 | 547479 | 496 | 187 | 6.8 | 4.31 | 220 | SRR33854600 |
| D87 |  | 04-09-23 | 06:00-14:00 | 8h | 11040 | 371713 | 371074 | 377 | 177 | 6.4 | 4.35 | 231 | SRR33854597 |
| D88 |  | 04-13-23 | 06:00-14:00 | 8h | 11040 | 481582 | 480828 | 750 | 260 | 5.7 | 4.93 | 360 | SRR33854596 |
| D89 |  | 05-10-23 | 14:00-22:00 | 8h | 11040 | 579263 | 578384 | 1134 | 270 | 9.8 | 4.75 | 363 | SRR33854595 |
| D90 |  | 05-12-23 | 06:00-14:00 | 8h | 11040 | 357535 | 356978 | 727 | 245 | 8.9 | 4.97 | 304 | SRR33854594 |
| D91 |  | 05-12-23 | 14:00-22:00 | 8h | 11040 | 517531 | 516653 | 258 | 107 | 4.3 | 2.91 | 148 | SRR33854592 |
| D92 |  | 05-13-23 | 06:00-14:00 | 8h | 11040 | 486694 | 485895 | 462 | 165 | 5.3 | 3.68 | 205 | SRR33854591 |
| D30 | Monsoon (n=23) | 06-03-22 | 14:00-22:00 | 7h 20m | 10120 | 379394 | 378963 | 280 | 117 | 2.7 | 3.15 | 135 | SRR33854567 |
| D31 |  | 06-03-22 | 22:00-06:00 | 8h | 11040 | 459677 | 459096 | 246 | 100 | 2.5 | 3.48 | 118 | SRR33854565 |
| D32 |  | 06-04-22 | 06:00-14:00 | 7h 35m | 10465 | 126135 | 125974 | 252 | 103 | 2.6 | 3.83 | 105 | SRR33854564 |
| D33 |  | 06-04-22 | 14:00-22:00 | 7h 50m | 10810 | 374044 | 373564 | 640 | 192 | 3.7 | 4.78 | 254 | SRR33854563 |
| D34 |  | 06-05-22 | 14:00-22:00 | 7h 30m | 10350 | 526100 | 525450 | 332 | 124 | 2.9 | 2.69 | 165 | SRR33854562 |
| D35 |  | 06-05-22 | 22:00-06:00 | 8h | 11040 | 340403 | 340013 | 403 | 129 | 3.2 | 4.09 | 152 | SRR33854561 |
| D36 |  | 06-06-22 | 06:00-14:00 | 8h | 11040 | 280162 | 279830 | 637 | 216 | 3.9 | 5.18 | 271 | SRR33854560 |
| D37 |  | 06-06-22 | 22:00-06:00 | 8h | 11040 | 377305 | 376663 | 502 | 204 | 6.2 | 4.87 | 258 | SRR33854559 |
| D38 |  | 06-07-22 | 06:00-14:00 | 8h | 11040 | 268179 | 267802 | 409 | 156 | 4.2 | 4.68 | 165 | SRR33854558 |
| D39 |  | 06-07-22 | 14:00-22:00 | 7h 40m | 10580 | 366860 | 365949 | 153 | 59 | 2.9 | 2.38 | 53 | SRR33854557 |
| D40 |  | 06-07-22 | 22:00-06:00 | 8h | 11040 | 274146 | 273747 | 209 | 81 | 2.5 | 3.4 | 95 | SRR33854556 |
| D41 |  | 06-08-22 | 06:00-14:00 | 8h | 11040 | 293899 | 293469 | 216 | 103 | 2.6 | 2.89 | 90 | SRR33854554 |
| D42 |  | 06-08-22 | 14:00-22:00 | 7h 50m | 10810 | 421030 | 420424 | 376 | 124 | 3.4 | 2.08 | 128 | SRR33854553 |
| D43 |  | 06-08-22 | 22:00-06:00 | 8h | 11040 | 154817 | 154653 | 199 | 71 | 3.4 | 3.42 | 83 | SRR33854552 |
| D44 |  | 08-18-22 | 13:00-22:00 | 6h 32m | 9016 | 312477 | 312104 | 216 | 81 | 2.5 | 3.22 | 85 | SRR33854551 |
| D45 |  | 08-18-22 | 22:00-06:00 | 7h 03m | 9729 | 216915 | 216657 | 360 | 90 | 3.8 | 2.45 | 96 | SRR33854550 |
| D46 |  | 08-19-22 | 06:00-14:00 | 6h 23m | 8809 | 248444 | 248164 | 188 | 52 | 2.6 | 1.84 | 43 | SRR33854549 |
| D47 |  | 08-19-22 | 14:00-22:00 | 7h 15m | 10005 | 214840 | 214545 | 206 | 94 | 2.9 | 3.68 | 115 | SRR33854548 |
| D48 |  | 08-19-22 | 22:00-06:00 | 6h 10m | 8510 | 176507 | 176268 | 214 | 72 | 2.7 | 2.1 | 72 | SRR33854547 |
| D49 |  | 08-22-22 | 06:00-14:00 | 8h | 11040 | 889182 | 884686 | 540 | 190 | 4.6 | 2.13 | 268 | SRR33854546 |
| D93 |  | 06-06-23 | 06:00-14:00 | 8h | 11040 | 165836 | 165535 | 722 | 275 | 6.2 | 4.83 | 364 | SRR33854590 |
| D94 |  | 06-06-23 | 22:00-06:00 | 8h | 11040 | 373132 | 372396 | 155 | 73 | 3.1 | 2.92 | 105 | SRR33854589 |
| D95 |  | 06-07-23 | 14:00-22:00 | 8h | 11040 | 456464 | 455486 | 175 | 89 | 3.5 | 2.13 | 121 | SRR33854588 |
| D50 | Post-monsoon (n=15) | 09-17-22 | 06:00-14:00 | 8h | 11040 | 443395 | 442562 | 226 | 110 | 3.9 | 3.68 | 161 | SRR33854545 |
| D51 |  | 09-17-22 | 14:00-22:00 | 8h | 11040 | 861158 | 859512 | 720 | 237 | 7.3 | 3.67 | 328 | SRR33854543 |
| D52 |  | 09-17-22 | 22:00-06:00 | 8h | 11040 | 942464 | 940895 | 564 | 225 | 4.5 | 3.79 | 304 | SRR33854542 |
| D53 |  | 09-20-22 | 14:00-22:00 | 8h | 11040 | 366357 | 366356 | 231 | 123 | 3.8 | 4.12 | 165 | SRR33854541 |
| D54 |  | 09-22-22 | 14:00-22:00 | 8h | 11040 | 302215 | 301679 | 432 | 158 | 3.7 | 4.48 | 209 | SRR33854540 |
| D55 |  | 11-15-22 | 06:00-14:00 | 8h | 11040 | 423176 | 422479 | 790 | 274 | 4.3 | 4.55 | 319 | SRR33854539 |
| D56 |  | 11-15-22 | 14:00-22:00 | 8h | 11040 | 358074 | 357420 | 838 | 228 | 6.8 | 4.65 | 292 | SRR33854538 |
| D57 |  | 11-15-22 | 22:00-06:00 | 8h | 11040 | 470238 | 469202 | 332 | 154 | 3.6 | 4.24 | 179 | SRR33854537 |
| D58 |  | 11-17-22 | 14:00-22:00 | 8h | 11040 | 374051 | 373469 | 1171 | 260 | 7.8 | 5.05 | 324 | SRR33854536 |
| D59 |  | 11-17-22 | 22:00-06:00 | 8h | 11040 | 238504 | 237063 | 240 | 106 | 4.2 | 3.92 | 141 | SRR33854535 |
| D60 |  | 11-19-22 | 06:00-14:00 | 8h | 11040 | 358749 | 358168 | 454 | 177 | 4.8 | 4.29 | 229 | SRR33854534 |
| D61 |  | 11-19-22 | 14:00-22:00 | 8h | 11040 | 398676 | 397998 | 737 | 227 | 7.4 | 4.5 | 272 | SRR33854532 |
| D62 |  | 11-19-22 | 22:00-06:00 | 8h | 11040 | 163730 | 163382 | 248 | 107 | 3.9 | 2.79 | 132 | SRR33854531 |
| D96 |  | 09-28-23 | 06:00-14:00 | 8h | 11040 | 149736 | 149497 | 177 | 81 | 3.7 | 3.69 | 115 | SRR33854587 |
| D97 |  | 09-28-23 | 14:00-22:00 | 8h | 11040 | 307702 | 307179 | 224 | 104 | 4.1 | 3.92 | 129 | SRR33854586 |
